# Women’s knowledge of and attitudes towards group B streptococcus (GBS) testing in pregnancy: a qualitative study

**DOI:** 10.1186/s12884-023-05651-0

**Published:** 2023-05-11

**Authors:** Georgina Constantinou, Susan Ayers, Eleanor J Mitchell, Kate F Walker, Jane Daniels, Sarah Moore, Anne-Marie Jones, Soo Downe

**Affiliations:** 1 Centre for Maternal and Child Health, School of Health Sciences, City, University of London, Northampton Square, London, EC1V 0HB UK; 2 Nottingham Clinical Trials Unit, University of Nottingham, Nottingham, UK; 3 Population and Lifeorgdivision Unit, School of Medicine, University of Nottingham, Nottingham, UK; 4 School of Community Health and Midwifery, University of Central Lancashire, Preston, UK

**Keywords:** Group B streptococcus, GBS, Screening, Testing, Maternal colonisation

## Abstract

**Background:**

20–25% pregnant women in the UK carry group B streptococcus (GBS) which, if left undetected, is transmitted from pregnant mothers to their babies during birth in 36% of cases. This transmission leads to early onset GBS infection (EOGBS) in 1% of babies which is a significant cause of mortality and morbidity in newborns. The literature available suggests women’s knowledge of GBS is low, with many women unaware of the GBS bacterium. In addition, attitudes towards GBS testing have not been widely examined, with research mostly focusing on attitudes towards potential GBS vaccination.

**Aim:**

To examine women’s knowledge of GBS in pregnancy and their attitudes towards GBS testing.

**Methods:**

Semi-structured interviews with 19 women (5 pregnant and 14 postpartum). Interviews were transcribed and analysed using systematic thematic analysis.

**Results:**

Four main theme categories were identified. Participants had varying levels of awareness of GBS, with the information provided by health professionals not being clearly explained or the importance of GBS being downplayed. Participants wanted more information and to feel informed. Overall, the majority had positive attitudes towards being offered and taking up GBS testing, and this study identified some of the key factors influencing their decision. These included: seeing GBS testing as just another routine procedure during pregnancy; that it would lower the risk of their baby becoming unwell; provide reassurance; and allow them to prepare; and provide informed choices. Participants also expressed a few common concerns about GBS testing: questioning the invasiveness of the procedure; risks to themselves and the baby; and the risk of receiving antibiotics.

**Conclusions:**

Women need clear, detailed information about GBS and GBS testing, and women’s concerns are important to address if routine GBS testing is implemented. The efficacy of implementing routine universal testing in the UK is currently being investigated in a large multi-centre clinical trial; the GBS3trial, further qualitative research is needed to look at the acceptability of different methods of GBS testing, as well as the acceptability of GBS testing to women in specific groups, such as those planning a home birth or those from different ethnic backgrounds.

## Introduction

In the UK 20–25% of pregnant women are carriers of group B streptococcus (GBS) bacteria [1], which, if not detected, can be passed from the pregnant mother to the baby during birth. It is reported that mothers who are carriers of GBS have a 36% chance that their baby will also become colonised with the bacterium during labour [1]. For most babies, this GBS colonisation will be asymptomatic [1], however, around 1% of these infants will develop an invasive - GBS disease [1]. If this occurs in the first 6 days after birth this is known as an Early Onset GBS (EOGBS) infection, which is a leading cause of mortality and morbidity in newborn infants [2]. EOGBS infections are reported to have mortality rate in the UK estimated 10.6% and higher rates of case fatality among preterm infants [3]. These EOGBS infections typically present as pneumonia, sepsis or meningitis with evidence suggesting around 517 babies per year in the UK are affected by early-onset disease [3]. Research suggests there are 53 deaths accountable to GBS disease in the UK annually [3].

GBS testing enables clinicians to identify pregnant women who carry the GBS bacterium and who therefore have the potential to transmit GBS to their babies. Women can be tested for GBS during pregnancy, which involves vaginal and rectal swab specimens being taken for culture tests [4, 5]. However, the current approach to managing GBS detection in the UK relies on identifying women at high risk of GBS and offering those women intrapartum antibiotics. The RCOG green top guidelines 36 identified those maternal risk factors for GBS [6]. This practice means only those ‘higher risk’ women are offered intrapartum antibiotic prophylaxis (IAP) [6] to reduce the risk of babies developing EOGBS infection [7].

This practice in the UK is a result of the National Screening Committee’s (NSC) decision not to screen universally at present until more evidence is available [8]. The NSC made this decision due to concerns that: a woman may have a positive result a few weeks before labour and a negative result when she gives birth; GBS does not cause an infection in every baby; screening may result in women having antibiotics when they do not need them; not enough understanding of screening outweighing the harms for most of the population and that the proportion of babies affected by the disease in countries where screening is carried out is similar to that in the UK [8].

However, there is a discussion surrounding the effectiveness of a risk-factor-based strategy with evidence showing that as many as 71% of babies with EOGBS infection had no recognised maternal risk factors for GBS [9]. In addition, another study reported that of 429 UK cases of EOGBS, only 35% of mothers had one or more risk factors for developing the infection and therefore are identified as ‘higher risk’ [10].

The management of GBS testing varies internationally [5], with many high-income countries such as the United States of America (USA) providing routine universal testing. This testing approach has resulted in a reduction in EOGBS infection rates per 1000 live births falling from 0.47 in 1999–2001 to 0.25 in 2010 [11]. In contrast, in the UK, statistics show an increase in incidence from 0.48 to 1000 live births in 2000 [12] to 0.57 per 1000 live births in 2014 − 1015 [3]. The efficacy of implementing routine universal testing as opposed to risk-factor based approach in the UK is currently being investigated in a large multi-centre clinical trial; the GBS3trial see ISRCTN registration (https://www.isrctn.com/ISRCTN49639731).

Whilst work is being done to ascertain the efficacy of routine testing for GBS in pregnancy, an important part of considering the implementation of GBS testing in pregnancy is understanding women’s awareness and attitudes towards GBS testing. Evidence suggests women’s knowledge of GBS testing to be low, with some studies highlighting that many women are unaware of the GBS bacterium [13–16]. Attitudes and acceptability towards GBS testing have not been widely examined [17, 18], with studies mostly focusing on attitudes towards a potential GBS vaccine [19, 20]. While these studies often include an element of women’s knowledge of GBS they do not assess women’s attitudes towards being tested for GBS and potentially being offered IAP treatment. With the GBS3 Trial underway, women’s knowledge, awareness and attitudes towards testing must be explored to understand women’s views on whether they would be willing to receive these types of tests in the future. Qualitative research is therefore needed to explore and understand women’s views, attitudes, and preferences in more detail.

Ths research aimed to qualitatively examine women’s knowledge of GBS testing and understand their attitudes towards being tested for GBS during pregnancy. The results will provide the information needed to understand whether it is acceptable to introduce this type of testing for women, which will be important both for the GBS3 trial and future decision-making about the implementation of a GBS screening programme.

## Methods

### Design

A qualitative interview study examining women’s knowledge of GBS in pregnancy, their attitudes towards GBS testing and views on specific methods of testing i.e. self-swabbing versus clinician-swabbing. The study is reported in line with the COREQ reporting guidelines. Women’s views on the specific methods of testing are reported elsewhere (Constantinou et al., submitted).

### Ethical approval

Ethical approval for the study was obtained from City, University of London, School of Health Sciences Research Ethics Committee (Ref: ETH2021-0149).

### Sample

A convenience sample of women was recruited using study advertisements via social media network channels (e.g. Twitter, Instagram and Facebook). Participants were eligible if they were: currently pregnant or had a baby in the last 2 years; aged 16 years or older (no upper age limit); had adequate spoken English; and were able to give informed consent. There were no exclusion criteria.

### Procedure

Potential participants received information about the study via targeted advertisements (digital posters) distributed on social media sites. The study used an incentive to facilitate recruitment, in the form of a prize draw to win a £25 retail voucher (n = 2), for those who participated in the study. Prizes were drawn after the data collection was complete. Those who expressed an interest in taking part contacted the Research Assistant (GC), who then provided a study invitation letter and participant information sheet providing more detail. Once they had considered the information about the study, they were then given an opportunity to ask the researcher questions and decide if they would like to participate. Those interested in taking part were screened for eligibility. Those who wanted to participate were then provided with a study consent form which they were asked to sign and return to the researcher. The researcher then contacted the participant to reconfirm consent and arrange a convenient time for an interview. Participants were offered either telephone or video call interviews arranged at a mutually convenient time and carried out 1:1 by an experienced qualitative researcher (GC, female, PhD).

Semi-structured interviews were conducted using a topic guide designed to explore (i) women’s knowledge of GBS testing; (ii) attitudes towards being tested for GBS in pregnancy, and (iii) acceptability of testing methods including self-swabbing procedures. This topic guide was pilot tested before data collection commenced. Interviews lasted around 30 min. Phase one of the interview asked women questions about their knowledge and awareness of GBS. Before moving on to the second phase, the researcher provided summarised verbal information about GBS from the Group B Strep Support and Royal College of Obstetricians & Gynaecologists (RCOG) Patient Leaflet [21]. It is important to note the researcher did not have a clinical background, participants were aware of this. This approach was used to ensure all participants had information the same information about GBS and GBS testing methods before asking questions about their attitudes towards and the acceptability of this testing. At the end of the interview, participants were asked to provide basic sociodemographic information such as; age, ethnicity, if they had any previous GBS experience and whether this was their first pregnancy. Field notes were made throughout the data collection and analysis process to facilitate reflexivity and monitor the saturation of the experiences shared.

### Data analysis

Audio recordings were transcribed and de-identified before analysis. Transcripts were analysed using systematic thematic analysis [23, 24]. All transcripts were read to become familiarised with the data, and then re-read and coded until no further codes were identified. After this, the codes were examined by two researchers (GC and SA) to extract the most salient and frequent codes which could be integrated into main themes. Data were examined for confirming and disconfirming information for each theme. Interviews for pregnant and postpartum women were analysed together. Analysis was conducted using NVIVO12, qualitative analysis software [24].

## Results

### Participant characteristics

Participants’ characteristics are given in Table 1. Twenty-four women expressed an interest in participating and nineteen (79%) consented to take part. Reasons for drop out included lack of time to be interviewed. Participants were aged between 25 and 42 years. Six were pregnant at the time of taking part and thirteen were postpartum. Of the nineteen participants, six were pregnant with or just had their first baby. Three participants had experienced or intended to have a home birth. Despite attempts to recruit participants with a range of ethnicities by advertising the study through black and minority ethnic birthing groups on social media and tailoring the study adverts to represent non-white women so the images reflected the target group we wanted to reach, the participants were White British [18] and Asian British [1].

Two-thirds (68%) of participants were postpartum at the time of the interview. The average age was 32 (SD 4.6). Most participants had no prior experience with GBS, however, three (16%) had received diagnoses of GBS in their current or previous pregnancy. Five participants had prior experience with GBS, gained from friends and family or their profession. Each interview lasted approximately 24 minutes.

**Table 1:** Sample characteristics (N = 19)

Characteristic	N (%)
Pregnant Postpartum	6 (32) 13 (68)
**Ethnicity**	
White British Asian British	18 (95) 1 (5)
**Experience of GBS**	
Diagnosed with GBS Other experience of GBS (Friend or family with GBS, professional role) No experience	3 (16) 5 (26) 11 (58)
**Number of pregnancies**	
1 (first pregnancy) 2 or more	7 (37) 12 (63)

### Thematic analysis

Four main theme categories were identified: [1] Awareness of GBS; [2] Information about GBS and GBS testing; [3] Positive attitudes towards testing; and [4] Concerns about testing. Each theme had several subthemes which are shown in Table 2 and outlined in more detail below.

**Table 2:** Main themes

Categories of Themes	Themes
**1: Awareness of GBS**	Differing levels of awareness GBS not detected or clearly explained
**2: Information about GBS and GBS testing**	Women want more information Information is not forthcoming How information is given Need to feel fully informed
**3: Positive attitudes towards testing**	Just another procedure Lowers the risk Provides reassurance Allows women to prepare for what may happen Creates informed choices
**4: Concerns about testing**	Risks to mother and baby/ Invasiveness of tests Risks of antibiotics May cause unnecessary worry Pressure to agree Influence on birth experience

### Theme 1: Awareness of GBS

The first theme highlighted participants’ ***differing levels of awareness*** of GBS in pregnancy and factors that contributed to this awareness. Many of the participants in this study had never heard of GBS (n = 8). Three had heard of it but were unaware of what it was (n = 3).

*“Very little knowledge. I mean, I’d heard of it, and that was it, I don’t really know what it does or anything*”* (W14)*

Others had an understanding of GBS from their professional working role or had experiences of friends or family members who had experienced GBS.

*“So, I was aware of it before because my mum had it when she gave birth to my little brother*”* (W4)*

In addition, some participants had awareness from information or discussions in forums such as antenatal groups (e.g. NCT, homebirth), online forums, television, and social media (n = 5). These prompts encouraged them to find out more information for themselves.

*“Well, my partner’s mum, said “I saw this interview on TV the other day, this man whose child had this thing”. And she said what it was…and she’s like “And they don’t test for it and I think you should definitely pay for the test”…So, yes, all I know about it now, is stuff that I’ve researched in the last few months*”* (W16)*.

Three participants had the experience of being GBS positive in their current or previous pregnancy. Two participants also shared experiences of having an infection in a previous pregnancy which could have been GBS although it was not confirmed at the time. However, even those participants with experience of GBS in pregnancy had levels of awareness and understanding that differed and some participants said that GBS was **not detected or clearly explained** to them at the time.

*“...basically it was still to this day, do not know what was wrong, they just put [baby name] on a course of antibiotics. And what they said was suspected sepsis, but Group B Strep was mentioned, so that’s basically my knowledge*”* (W14)*

It was also discussed that awareness of GBS for one participant came from being diagnosed, at which time she was reassured that it was not a major concern and that she would receive antibiotics.

*“I wasn’t aware of it as anything, I’d never heard of it before I got pregnant with my first child, I was only aware of it because I came positive with it. My midwife said it’s fine, it’s nothing major*”* (W10)*

### Theme 2: Information about GBS and GBS testing

The majority of participants said they ***would like to be provided more information*** about GBS in pregnancy and felt it is important that parents have awareness and feel informed about GBS testing.

*“My advice would be is because I’d never heard of it, so before getting told I had it, my advice would be is to inform parents and make them aware of what the consequences are if you have it and you don’t take the antibiotics if you don’t get tested*”* (W10)*

Women also discussed why being provided with this information early is necessary to allow them to make decisions about treatments in a timely manner.

*“I guess, it would be like making sure that they had information early on, so they can make an informed decision, in a calm way and in their own speed, so, that it wasn’t just on the spot, there and then*”* (W16)*.

It was identified that some participants felt the ***information was not forthcoming*** unless they were already aware of GBS and asked for information themselves. Some also described this information as hidden, while others had experiences of healthcare professionals downplaying the importance of GBS.

*“It was very, not swept under the rug, but like an unheard of thing, because I know, from my own experience, unless I’ve mentioned it, it hasn’t been mentioned to me*”* (W4)*

*“Just to talk about it. It sounds a little bit like possibly it’s been hidden, or, well, has been hidden, possibly. Because I’ve never heard of it, and I’ve had a child recently. So I think that, yeah, talk about the risks*”* (W5)*

Some participants who asked about GBS were told: not to worry; that it is rare; and was something that was not tested for. As a result, they described feeling dismissed when seeking information.

*“she essentially gave us the impression that it’s so rare you don’t need to worry*”* (W3)*

Discussion of ***how information is given*** was felt to be important, particularly that midwives should not presume women are aware of important information nor that women do not need detailed explanations. Some participants experienced little explanation of the testing they had received and felt they thus had a lack of understanding of what may happen next.

*“I knew [about GBS], but I think also as a parent it would have been nice to have some leaflets or someone… to run through it with me again. It’s quite different when you’re going through it yourself as to being a midwife and being on the other side of it. No, I didn’t get any information. They put a sticker on my notes to say GBS alert but no, nobody’s actually run through anything with me apart from the fact that if my waters break I have to go straight in*”* (W9)*

Participants also shared a preference for receiving this information in person and it was deemed

important that it was provided in various ways i.e., written, verbal etc. to ensure this was accessible to a range of service users.

*“everyone responds to things differently. Like some people will read the written word…and then equally some people would want to have that conversation…and then being able to have back and forth and being able to ask questions, you can’t go wrong with having it written down, because then, you can go to it online or whatever, in a leaflet that you’re given, at any time that you want it. Then you don’t have to worry that you’ve forgotten the information or whatever. I think it makes it then more accessible to a wider range of women, whether that’s like, in terms of, language barriers or like, cognitive processing, things like that*”* (W16)*

Some reported the ***need to feel fully informed*** to make decisions about their care and this played a role in shaping their attitudes towards testing.

*“Probably offer clear information to people who are test positive for GBS so that woman feels fully informed and can make a proper decision on the care going forward I think*”* (W9)*

While participants shared their thoughts about the availability of information surrounding GBS, they also expressed their views about the testing itself and how this can be influenced by the understanding they have of GBS. The majority were not opposed to the idea of GBS testing in pregnancy but they had questions and would like to know what they are committing to before deciding whether or not they would like to be tested.

*“if I didn’t know what it was at all, then I would want in-depth information about what it is and what I was committing to*”* (W16)*

When discussing women’s attitudes towards testing in general, it was highlighted that some participants may be put off the idea of having GBS testing if they misunderstand what it entails.

*“I think perhaps it depends on what they think is involved. So, if they think it’s going to be like a smear test and having a speculum and everything like that, they might be put off*” *(W1)*

Overall, the majority of participants interviewed said they would have a test if offered and only one participant felt she would not accept testing. Most were also keen to share information about GBS with a friend or family member and said they felt it was important to recommend testing. The participants that were interviewed felt that the most effective way to make GBS testing more acceptable to women would be by offering it routinely to everyone. There would then be an increased familiarity with matters relating to GBS and EOGBS infection. It was felt strongly that information from health care professionals should clearly explain that being tested for GBS remains a matter of choice and is not compulsory.

*“I would say offer it to everybody. Yes, exactly, if you want to take it, wonderful, if you don’t also that’s fine. I think the offer should be there*”* (W13)*

### Theme 3: Positive attitudes towards testing

Theme three identifies participants’ positive attitudes towards GBS testing. Most participants described being offered GBS testing as **just another procedure**, viewing this as a simple test which they would be happy to receive. Participants described having so many other tests and procedures during pregnancy that one additional test would not be a concern for them.

*“I mean the thing is you have blood tests anyway, you’re already getting other things. I went in with [baby name] and had to have them check me and all that kind of stuff, so just taking a quick swab is not going to make a difference really, is it?*”* (W13)*

The use of swabs and intravenous antibiotics was viewed as simple and important to protect against the risks of GBS posed to the baby.

*“if it could be fatal towards your baby and it can be cured by just a simple test and putting an antibiotic drip up, I think it is really important*”* (W11)*

It was discussed that GBS testing is less invasive than some other types of screening in pregnancy and that participants would perhaps thus be more likely to agree to GBS testing.

*“To be honest, I don’t know, how other people would feel, but I would say that 80 percent of mums just nod and agree to whatever ... unless it’s something that’s extremely intrusive, there are some tests in pregnancy where…they want to insert needles, or things, into the womb. That is very different*”* (W12)*

Even though GBS swabbing was perceived to be more intimate than a blood or urine test, some participants felt that they had to undergo other undignified procedures in labour, and therefore would be accepting of this type of testing.

*“because it is quite invasive, it’s vaginal or rectal, but you’re about to have a baby and you’re going to lose all your dignity there*”* (W13)*.

Some participants said they would be willing to accept any test as long as there was no financial or physical cost to themselves or the baby.

*“I think any test that I’ve been offered, like, when I’ve had bloods taken and stuff, if it’s not going to be any extra [financial cost], if it’s not going to hurt me or the baby then I think why wouldn’t I*”* (W3)*.

Some discussed the balance of risk and benefits, concluding in favour of testing:

*“Obviously it does come to a cost to the NHS, per pregnant lady, which will obviously add up, and will be quite costly over time. But actually if you’re saving lives, and also children who, like you said, could have significant difficulties growing up, actually when you weigh up how much that would potentially cost in support that would be required for those children anyway, actually yeah I think it … I think it’s a no brainer*”* (W12)*.

Many participants recognised the value in testing, despite there being only a small GBS risk, due to potential of the any baby dying which could have been avoided.

*“I think some people might worry about it unnecessarily if you get so many people that are unaffected and babies that are unaffected, so it could cause unnecessary worry but I would rather have that worry and peace of mind, even if it’s a really small statistic, if that’s going to save one baby’s life, to me I think that’s worth it*”* (W3)*

This prevention was also felt to be critical with ***lowering the risk*** important to participants:

*“If there’s a way that you can prevent it, it’s better to be preventative than to wait for something to develop and then have to react to it. Yeah, so I think you should be tested*”* (W4)*.

For many participants, pregnancy created an instinctive feeling of responsibility to protect their baby. This was used to explain their attitude towards wanting the GBS testing, with participants questioning why other women would not want to seek testing if it can prevent harm.

*“I think if it’s not putting the mum at risk, it’s not putting the baby at risk but it could potentially prevent a serious illness, for me that’s a no brainer, if it saves one life a year by doing it that’s worth it to me*”* (W3)*

*“Again, purely the fact that if there’s that tiny chance, I’m the type of person that just wouldn’t take the chance, so I’d be saying to whoever it was… to that other person it might only be a really small chance, but nothing is worth that risk*”* (W13)*

It was also expressed that women have a right to know about any potential risk to their baby.

*“I just think everyone has the right to know and they should, it’s their baby at the end of the day, I know it’s a very unlikely chance that something could happen but there’s still that possibility*”* (W19)*

Many respondents implied that testing would ***provide reassurance*** and peace of mind, even if it was not 100% accurate:

*“A lot of testing isn’t one hundred percent, I think we accept that, so I think if the NHS and medical professionals are worried about causing people to worry or give false results, it’s kind of the same in every situation, I think most people understand that things aren’t a hundred percent guaranteed but it’s nice to have that reassurance*”* (W3)*

The benefits of a positive test was discussed in terms of **allowing women to prepare** for what may happen next:

*“I think pros would be having that peace of mind, even if you were positive obviously it might cause a bit of anxiety but at least you know it can be resolved and you can do something about it, it’s not going to be a shock to the system later down the line*”* (W3)*

This included preparing to make a decision as to whether they would want treatment for GBS if they tested positive and what would happen in this circumstance. While also psychologically preparing for potential risks that it could cause for the baby if it is transmitted to them.

*“Just so then I know, I can then think about the antibiotics if I need them and the baby if the baby needs them, it does give me chance to think about it instead of just being thrown in that situation*”* (W19)*

Surprisingly, only a few participants observed that testing allowed for early treatment:

*“Yes, anything really can be tested for as a positive, if it gets caught early and it’s more treatable, then I think it’s worth doing" (W15)*

*"That it could be treated, sooner rather than later*”* (W8)*

The majority of respondents felt that the offer of testing ***creates informed choices***.

*“I just feel that we should always be offered as many things as we can, and so much of like, antenatal care, is an offer…So, like what’s the harm in being offered another test that could give you some information that might potentially, save your baby’s life*”* (W16)*

Some participants felt that the cost of treating a woman with antibiotics would be less than treating a baby for EOGBS and therefore had a positive attitude towards the cost of testing.

*“to give out antibiotics to women who have it, it’s probably going to be cheaper…, than treating a baby who’s got meningitis or whatever. With everyone’s increasing resistance to antibiotics,…providing antibiotics at such an early stage for a baby and for a woman that might not necessarily need it, I guess, there’s always downsides, but I feel like, those aren’t equivalent to the potential risks of the baby having a really serious infection and dying*”* (W16)*

### Theme 4: Concerns about testing

Theme 4 captured participants’ concerns surrounding being offered GBS testing, many of which were focused on the risks the testing posed to them, and, particularly, ***how invasive the procedure would be.*** This encompassed whether this would be uncomfortable or distressing for women. A common misunderstanding of what the swabbing involved was that the swab used a High Vaginal Swabbing (HVS) technique which involved a speculum with some comparing it to a smear test:

*“I think it’s quite invasive and most of the time you have to have a speculum which is very uncomfortable and maybe the HVS which is a high vaginal swab so it goes up quite deep inside the vagina. It can just, some people might find it traumatic*”* (W9)*

Some participants were concerned about potential ***risks posed to their baby*** if they agree to have GBS testing, with concerns expressed that a test late in pregnancy may induce early labour.

*“It would definitely be something I would ask, only because I know that late on, even something as small as… express[ing] milk early on. And that I know can induce early labour…I would wonder whether, it would just make me question whether vaginal swab would do that*” *(W5)*

Alongside potential risks to the pregnancy, participants also expressed their ***concerns about needing antibiotic treatment*** if they test positive for GBS. It was indicated that they would want reassurance that the antibiotics offered would be both safe for their baby and would minimize the risk of EOGBS.

*“I’d say as long as they’re safe and not going to harm my baby I’ll take them because as you mentioned the risk is there. I’d prefer to take antibiotics and the risks not be there anymore*”* (W11)*

Participants also shared concerns as to whether antibiotics would affect them being able to breastfeed their baby, or about the potential impact on their own wellbeing, especially if the antibiotics were actually unnecessary.

*“The risk of antibiotics to breastfeeding, I think that would be something that would come up to mums. Because they would ask you straightaway, “Can I feed my baby because of this antibiotic?” And if you’ve got a person who’s having a swab done in early labour, and they found out that they’ve got it, and then the mum straightaway was put onto the antibiotics, that mum would probably invariably be afraid to feed her baby, because of the antibiotics. And wouldn’t have had time for that to have been discussed with her*”* (W5)*

A concern discussed frequently (n = 10) by the participants was whether offering testing ***would cause women to feel stressed, worried, be fearful, anxious or upset.*** Some participants felt that women may prefer not to know if they had GBS if it could cause them added stress at a late point in their pregnancy.

*“Whereas some people don’t want to know, because that then adds more stress to them, so that could be a considering factor, if … especially, at that late stage of pregnancy as well, like, if you were to be tested and it come back positive, then that might be an added stress*”* (W4)*

Participants said that being appropriately informed by their health care professionals about the risks of GBS would mitigate the risk of undue anxiety caused by testing.

*“But I think they just need to explain the risks a bit more… it’s really difficult because there’s a fine line between scaring a pregnant woman, and actually saying yeah, this could be a thing, this … I just don’t think there’s enough information*”* (W14)*

The timing of information provision regarding GBS was also considered important.

*“I would worry like if I was 35 weeks pregnant, and I had that test, I would be terrified…of going into labour and potentially my baby having issues or possibly dying. I gave birth to her at 41 weeks, so I would have had six weeks of worry*”* (W7)*

The ***pressure to agree*** to be tested was also discussed. Concern was expressed that women may only agree to testing because they feel they must in the circumstances, rather than it being truly informed consent.

*“it might be one of those things they don’t want to have a swab, but it’s like, “Well, you know, it’s for the best.” So, it’s not quite consent but it’s kind of, “Oh, it’s for the best. I don’t really want to” (W6)*

Concern was also raised in relation to why the woman was being offered the testing. It was discussed that some participants would fear that the professional may know something they do not about their risk of carrying GBS and this would worry them.

*“I would want to know why they’re asking me to do it, to be honest with you, I would want to, like, “Am I in one of the risk groups? Has something come up in my urine?” (W6)*

Potential anxiety created by GBS testing was also considered in relation to the relatively low number of women who would test positive and the even lower number of women who go on to be impacted by EOGBS, with some participants (n = 2) viewing the testing as a waste of time and the highlighting the imbalance between numbers actually affected and the numbers of women exposed to increased anxiety:

*“I would say that the numbers don’t sound huge in terms of those that are affected, but then actually, I know this is just a relative comparison at the moment, but in terms of how much fear has been placed into pregnant mothers at the moment*”* (W12)*

For some participants, particularly those who had previously given birth at home or had clear views on the birth they wanted, discussion of how GBS testing and IV antibiotics would ***influence their birth experience*** was raised. Some respondents were worried that this may stop them having the birth they had planned. This was especially true for those women who did not want to birth in a hospital setting or wanted a water birth (n = 3).

*“I would be asking the questions, like, “Can I have this at my home birth? What can I do to still have the birth that I want and manage this?”. So, in terms of a home birth and birth preferences and like managing my mental health, and physical state, it would be trying to be really organised and make sure that all bases were covered for me for getting the treatment in, making the baby safe…definitely information on how that would impact on the birthing place, or like the birthing process…and like risks*”* (W16)*

These respondents (n = 3) would like more information as to whether they would have to go to hospital if they tested positive, or if the IV could be provided at home. Although this is not an available option, these women explained the ideal that if this could be provided at home it was felt that they would be more willing to have the testing. IVs potentially restricting the women’s movement during labour was also an important factor considered. Additionally, some women felt that the benefits of being in the familiar home setting, and avoiding hospital pathogens, may outweigh the risks of GBS.

*“When you’re at home, you’re exposed to less bacteria and viruses and things like that, or ones that you’re used to, your body is already used to them. So, therefore, your baby is already used to them. So, it would be like weighing up all of those things and especially thinking about it in terms of, wanting to have a home birth, that there’s all of the really physical benefits to having it, at home, that could potentially, like outweigh the risks [of GBS]*”* (W16)*

A few participants were thinking beyond the risk of infection itself, taking into account the wider risks of hospital birth. This could be characterized as a balance between the odds of severe adverse effects of GBS and those of over-medicalising birth. One participant suggested that the ideal situation for women who want a home birth would be to have access to testing and then being able to receive the antibiotics at home if needed, ensuring benefit for the baby and good birth experience for the mother.

*“In an ideal world…I would do universal screening so everyone’s happy. But I would also implement it so we can give these antibiotics to women wherever they want. Wherever they want to give birth…because then you’re getting the benefits of the baby, but you’re also getting the benefits of the woman having a good birth experience*”* (W6)*

## Discussion

### Summary of findings

This study aimed to explore women’s knowledge of group B streptococcus in pregnancy, their attitudes towards GBS testing. Women had varying levels of awareness of GBS in pregnancy, with 58% saying they had never heard of it. While many of the women interviewed had never heard of GBS or were unsure of what GBS was, some had experience of being GBS positive in pregnancy and a couple of women had experienced infections during pregnancy which could have been GBS. Interestingly, awareness and understanding of GBS also varied within the women who had GBS, with some reporting GBS was not detected or clearly explained at the time.

Several factors contributed to women’s awareness of GBS. These included: friends or family experiences; experiences from their working role; forums; antenatal education groups; and the media. This is consistent with previous studies that had reported that participants had often gained knowledge about GBS through doctors, family, social media, work, television, friends, online research [25].

Women wanted more information about GBS. Some, including those who were GBS positive, reported being told by health professionals that GBS is rare, not tested for, and therefore they did not need to worry about it. Some reported that these experiences made them feel their concerns were dismissed. The way information was delivered was important, being informed early to facilitate decision making about treatment was essential for some. They wanted to know what they were committing to and to have their questions answered before agreeing to GBS testing. Previous research looking at GBS vaccines [26] has shown that even small amounts of knowledge about GBS, is effective in increasing willingness to have a GBS vaccine. This may also be true for willingness to be tested and to receive treatments if testing is to be routinely offered in the future, though this hypothesis would need to be tested with a larger and representative sample.

Respondents wanted to the information to be explained by health professionals who could discuss it with them and answer their questions, in line with previous studies [21]. However, the experiences of women in the current study suggest that health professionals do not always provide sufficient information. A study which explored physician awareness found that, although they had good knowledge of GBS, half wanted additional training about testing [25].

The majority of women had positive attitudes towards the offer of GBS testing. It was seen as ‘just another procedure’ that was part of pregnancy. Most thought that such an offer increased informed choice, allows preparation in case of a positive result and it would lower the risk of their baby being affected by EOGBS because it could be managed effectively. A few respondents, expressed concerns about testing questioning the invasiveness of the procedure and whether there were risks to themselves or the baby. As well as the possible iatrogenic effects of receiving prophylactic antibiotics. These concerns are very similar to those found in research looking at women’s views on a GBS vaccine [24]. In addition, there were concerns both that the offer might increase anxiety in women who were subsequently found to be negative or that some may feel clinical and social pressure to agree to being tested when they would prefer not to. For those women who had planned or had water births or home births there was concern that GBS testing might affect their choice of type of birth and place of birth (e.g. home, midwifery-led unit, hospital), and, consequently, increase risks of iatrogenic harm for them or their babies.

Overall, nearly all the women in this study said they would accept GBS testing if offered it; only one woman in this study said they would not accept testing. This is similar to research on womens’ attitudes towards potential vaccines for GBS [21] which shows the majority of women (85%) would be willing to accept a GBS vaccine if it would protect their baby from illness [21]. Most women in the current study felt GBS testing was important and would be keen to share information with friends and family. They suggested the best way to increase the acceptability of testing for GBS was to make the offer a routine part of care. Women thought that where GBS testing is routinely offered it would increase awareness, willingness and attitudes towards having testing and treatment. This is broadly consistent with research on attitudes towards GBS vaccines which found women relied on health professionals opinion and guidance to inform their decision making [24]. However, women in the current study felt strongly that testing should be authentically offered as a choice that could be declined, and that this should be made very clear to women.

For those who were concerned about the impact on place or mode of birth and consequent iatrogenic harm, the potential to have IV antibiotics at home was an important driver.

### Implications for practice and research

Women’s experiences suggested it is important that women’s and health care professionals awareness of GBS in pregnancy is raised, to enable women to have discussions about GBS with HCPs and therefore make an informed choice about testing in the future. While the majority of women were positive about being offered testing for GBS they also had reservations about ensuring they received enough information to make an informed decision. Thus, clear and detailed information about GBS and GBS testing needs to be provided to women in pregnancy, especially if the offer of routine testing is being implemented. Many of the concerns women reported in this study could be resolved by ensuring women have the correct information such as leaflets or videos about GBS and its testing. Those available to women include the GBSS/RCOG patient leaflet [22], which is the leaflet recommended by RCOG GTG to be provided by HCPs to women [6]. Although this is the recommended information that should be provided, this study suggests the leaflet may not be being utilized. Training for health professionals about how best to provide clear information to women in a way that aims to not cause unnecessary worry is important, particularly when women are accessing information about GBS through forums, antenatal groups and social media which are not guided or controlled sources of information. Enabling IV antibiotics to be given at home, or in ways that minimise interference with physiological labour and birth, are also likely to increase agreement to testing, and uptake of treatment where necessary. Further research is needed examining health professionals experiences of GBS, GBS testing and information provision as well as the barriers that may prevent them providing detailed information to women. In addition, if an offer of GBS testing is to be routinely implemented it would be important to look at the acceptability of different types of GBS testing methods to women.

### Methodological limitations

A key methodological limitation was that the sample was relatively small and, despite attempts to recruit a representative sample, the sample were predominantly White British. Previous literature has shown there are differences between White and Asian women on the topic [17], therefore more research is needed to determine the awareness and attitudes towards GBS testing from the perspective of women from other ethnic groups [17]. In addition, nearly half of the women had some awareness of GBS or had been diagnosed with GBS previously which may influence their attitudes or motivations towards the GBS testing and the topics discussed. Therefore, further qualitative studies with a larger more ethnically diverse sample are needed, as well as examination of women with and without experience of GBS.

Finally, more women in the study were postpartum rather than pregnant. Postpartum women will be considering GBS testing retrospectively – talking about what they would have decided if testing was offered. It is possible these views are different to how they might have thought when they were pregnant. However, by including both pregnant and postpartum women in the sample, both perspectives have been accounted for.

## Conclusion

This study provides an in-depth understanding of the respondents’ awareness of GBS and their attitudes towards being offered GBS testing during pregnancy. Their knowledge of GBS was limited and varied, even in those previously diagnosed with GBS during pregnancy. Their experiences suggest that information on GBS might not be provided by health professionals, and that, even when it is, it may not be clear or detailed enough. The respondents wanted more information to be provided to them to allow them to make informed decisions. Overall, the majority of women had positive attitudes towards being offered and taking up GBS testing, though there were some key caveats and concerns that might influence decision making However, the sample was not representative of the diversity of childbearing women. Further research is needed to look at the acceptability of different methods of GBS testing for all women, as well as for those not represented in this study, and to identify solutions for those who want to integrate testing and treatment with choices for home, waterbirth, and physiological labour and birth processes.

## Data Availability

The data generated and analysed during the current study are not publicly available as women did not consent to their data being publicly available but instead agreed to data being available from the corresponding author on reasonable request.

## List of abbreviations

GBS: Group B Streptococcus
EOGBS: Early Onset Group B Streptococcus
IAP: Intrapartum Antibiotic Prophylaxis
RCOG: Royal College of Obstetrics and Gynaecology
NIHR: National Institute for Health Research
HCP: Health Care Professionals
